# Italian Guidelines for the Management of Prolactinomas

**DOI:** 10.2174/1871530323666230511104045

**Published:** 2023-09-15

**Authors:** Renato Cozzi, Renata Simona Auriemma, Ernesto De Menis, Felice Esposito, Emanuele Ferrante, Giuseppe Iatì, Diego Mazzatenta, Maurizio Poggi, Roberta Rudà, Fabio Tortora, Fabio Cruciani, Zuzana Mitrova, Rosella Saulle, Simona Vecchi, Michele Basile, Paolo Cappabianca, Agostino Paoletta, Enrico Papini, Agnese Persichetti, Irene Samperi, Alessandro Scoppola, Alessandro Bozzao, Marco Caputo, Francesco Doglietto, Francesco Ferraù, Andrea Gerardo Lania, Stefano Laureti, Stefano Lello, Davide Locatelli, Pietro Maffei, Giuseppe Minniti, Alessandro Peri, Chiara Ruini, Fabio Settanni, Antonio Silvani, Nadia Veronese, Franco Grimaldi, Roberto Attanasio

**Affiliations:** 1 Department of Endocrinology ASST Grande Ospedale Metropolitano Niguarda, Milan, Italy;; 2 Department of Molecular and Clinical Endocrinology and Oncology, Federico II University of Naples, Italy;; 3 Internal Medicine 2, Treviso Hospital, Treviso; Functional Department of Endocrinology and Metabolism, AULSS 2 Veneto, Italy;; 4 Neurosurgical Clinic, Department of Neurosciences and Reproductive and Odontostomatological Sciences, “Federico II” University of Naples, Italy;; 5 Endocrinology Unit, Fondazione IRCCS Ca’ Granda Ospedale Maggiore Policlinico, Milan, Italy;; 6 Department of Radiation Oncology, University of Messina, Italy;; 7 Department of Biomedical and NeuroMotor Sciences (DIBINEM), University of Bologna, IRCCS Istituto delle Scienze Neurologiche di Bologna, Programma Neurochirurgia Ipofisi - Pituitary Unit, Bologna, Italy;; 8 Endocrinology, Department of Clinical and Molecular Medicine, S. Andrea Hospital, Sapienza University of Rome, Italy;; 9 Division of Neurology, Castelfranco Veneto and Treviso Hospital, Treviso; Division of Neuro-Oncology, Department of Neuroscience “Rita Levi Montalcini”, University of Turin, Italy;; 10 Radiology Unit, Department of Advanced Biomedical Sciences, University “Federico II”, Naples, Italy;; 11 Department of Epidemiology, Lazio Region Health Service, Rome, Italy;; 12 High School of Economy and Management of Health Systems, Catholic University of Sacred Heart, Rome, Italy;; 13 Endocrinology, ULSS6 Euganea, Padova, Italy;; 14 Endocrinology, Ospedale Regina Apostolorum, Albano Laziale;; 15 Ministry of Interior - Department of Firefighters, Public Rescue and Civil Defense, Rome, Italy;; 16 Endocrinology, ASL Novara, Italy;; 17 Endocrinology, Ospedale Santo Spirito, Rome, Italy;; 18 Neuroradiology, S. Andrea Hospital, NESMOS Department (Neuroscience, Mental Health, Sensorial Organs), Sapienza University of Rome, Italy;; 19 Laboratorio Analisi Cliniche e Microbiologia, Synlab SRL, Calenzano (FI), Italy;; 20 Institute of Neurosurgery, Catholic University School of Medicine, Rome, Italy;; 21 Department of Human Pathology of Adulthood and Childhood “G. Barresi”, University of Messina, Messina, Italy;; 22 Department of Biomedical Sciences, Endocrinology Unit, Humanitas Clinical and Research Center IRCCS, Humanitas University, Rozzano (MI), Italy;; 23 General Practitioner, USL Umbria 1, Perugia, Italy;; 24 Department of Woman and Child Health and Public Health, Institute of Obstetrics and Gynecology, Fondazione Policlinico Universitario Agostino Gemelli IRCCS, Rome, Italy;; 25 Division of Neurosurgery, Department of Biotechnology and Life Sciences, University of Insubria, ASST Sette Laghi, Varese, Italy;; 26 Department of Medicine (DIMED), 3^rd^ Medical Clinic, Padua University, Italy;; 27 Radiation Oncology Unit, Department of Radiological Sciences, Oncology and Anatomical Pathology, Sapienza University of Rome, Policlinico Umberto I, Rome, Italy;; 28 Pituitary Diseases and Sodium Alterations Unit, Endocrinology, AOU Careggi, Department of Experimental and Clinical Biomedical Sciences “Mario Serio”, University of Florence, Italy;; 29 Department of Psychology, University of Bologna, Italy;; 30 Clinical Biochemistry Laboratory, City of Health and Science University Hospital, Turin, Italy;; 31 Department of Neuro-Oncology, Fondazione IRCCS Istituto Neurologico Carlo Besta, Milano, Italy;; 32 AME past President, Udine, Italy;; 33 AME Scientific Committee, Milan, Italy

**Keywords:** Prolactinoma, prolactin-secreting tumor, microprolactinoma, macroprolactinoma, cost-efficacy analysis, cabergoline, bromocriptine, neurosurgery, radiotherapy, temozolomide

## Abstract

**Introduction:**

This guideline (GL) is aimed at providing a reference for the management of prolactin (PRL)-secreting pituitary adenoma in adults. However, pregnancy is not considered.

**Methods:**

This GL has been developed following the methods described in the Manual of the Italian National Guideline System. For each question, the panel appointed by Associazione Medici Endocrinologi (AME) has identified potentially relevant outcomes, which have then been rated for their impact on therapeutic choices. Only outcomes classified as “critical” and “important” have been considered in the systematic review of evidence and only those classified as “critical” have been considered in the formulation of recommendations.

**Results:**

The present GL provides recommendations regarding the role of pharmacological and neurosurgical treatment in the management of prolactinomas. We recommend cabergoline (Cab) *vs*. bromocriptine (Br) as the first-choice pharmacological treatment to be employed at the minimal effective dose capable of achieving the regression of the clinical picture. We suggest that medication and surgery are offered as suitable alternative first-line treatments to patients with non-invasive PRL-secreting adenoma, regardless of size. We suggest Br as an alternative drug in patients who are intolerant to Cab and are not candidates for surgery. We recommend pituitary tumor resection in patients 1) without any significant neuro-ophthalmologic improvement within two weeks from the start of Cab, 2) who are resistant or do not tolerate Cab or other dopamine-agonist drugs (DA), 3) who escape from previous efficacy of DA, and 4) who are unwilling to undergo a chronic DA treatment. We recommend that patients with progressive disease notwithstanding previous tumor resection and ongoing DA should be managed by a multidisciplinary team with specific expertise in pituitary diseases using a multimodal approach that includes repeated surgery, radiotherapy, DA, and possibly, the use of temozolomide.

**Conclusion:**

The present GL is directed to endocrinologists, neurosurgeons, and gynecologists working in hospitals, in territorial services or private practice, and to general practitioners and patients.

## 1 INTRODUCTION

### 1.1 Epidemiology

Prolactinomas are the most common pituitary adenoma, accounting for approximately 50% of all pituitary adenomas, with a prevalence of ∼50 per 100,000 population and an incidence of 3-5 new cases/100,000/year [1, 2]. Based on tumor size, they are classified as microprolactinomas (microP, < 10 mm diameter) or macroprolactinomas (MP, ≥ 10 mm diameter). Giant tumors (> 40 mm) are rare.

MicroPs are mainly observed in premenopausal women, whereas MPs are more common in men aged more than 50 years.

In a few cases, other pituitary hormones, mostly GH, are cosecreted in excess beyond PRL. PRL-producing pituitary carcinomas are rare and defined by the presence of metastases [3]. Prolactinomas can also occur in the context of genetic syndromes (1.5-3% of cases), mainly in multiple endocrine neoplasia type 1 (MEN-1) [4] and familial isolated pituitary adenoma (FIPA) [5]. Prolactinomas are not associated with increased prevalence of diabetes, cardiovascular diseases, or cancer, but premature mortality has been reported in patients bearing MP [6] probably due to hormonal deficiencies or their overtreatment.

### 1.2 Clinic

The patient may seek medical evaluation complaining of different symptoms, according to gender and age. Women of reproductive age complain of endocrine symptoms, such as oligo-amenorrhea or short luteal phase, decreased libido, anovulatory infertility, or galactorrhea [1, 7]. Postmenopausal women usually present with symptoms due to mass effects related to a large tumor. Approximately half of the males typically complain of signs and symptoms caused by the tumor mass and the other half by hypogonadism [1], most frequent of those being a loss of libido, erectile dysfunction, infertility and, less frequently, gynecomastia and galactorrhea [8]. Due to hypogonadism, men may also complain of decreased energy, impaired muscle mass and strength, and anemia [9].

Patients with MPs may seek medical attention due to mass effect signs and symptoms, such as visual impairment (mainly visual field defects), headache [10-12], and hypopituitarism. This condition may be due to direct compression of the adenoma on normal pituitary tissue, hypothalamic disconnection from stalk compression or, rarely, apoplexy [8]. Cranic nerve palsies, hydrocephalus, and skull base bone erosion are rare and late occurrences [13]. Hyperprolactinemia per se or through hypogonadism may result in osteoporosis and fractures [14, 15]. Effective treatment of hyperprolactinemia can restore normal bone mineral density [16].

### 1.3 Diagnosis

PRL secretion is pulsatile, and its serum levels are physiologically higher during sleep and in the early morning [17]. The diagnosis of hyperprolactinemia is established by measuring basal PRL levels. Emotional stress, venipuncture, exercise, and walking stimulate PRL secretion. Thus, specimens collected after an overnight fast, at least two hours after awakening and while the patient is resting, provide the most reliable PRL level assessment. A saline intravenous infusion through a G22 catheter, started 15-20 minutes before PRL sampling, prevents the use of a tourniquet and is a simple and reliable tool in cases of mild hyperprolactinemia [18]. It is advisable to use a 3-way stopcock and to discard the first 3 mL of blood that is diluted by the infused saline.

PRL assays typically employ noncompetitive, heterogeneous “sandwich” techniques with the use of two antibodies that recognize different epitopes on the PRL polypeptide. PRL methods should be calibrated against reference materials with known international unit potency, such as the WHO's first IRP 75/504, the second international standard (IS) 83/562, or the third IS 84/500. Attention must be paid to potential interferences on PRL assessment, especially when the clinical picture and results are discordant:

• Falsely high PRL levels may result from macroprolactinemia, a condition that is characterized by the predominance of big–big PRL in the serum. Polyethylene glycol serum precipitation is the most effective and inexpensive method for screening the presence of serum macroprolactin. Recoveries of PRL levels <40% are indicative of a macroprolactin predominance, which is less bioactive than the monomeric PRL and usually accounts for less than 1% of total serum PRL. Conversely, recoveries >60% suggest the diagnosis of monomeric true hyperprolactinemia [19-21].

• Artificially low PRL levels may result from the so-called ‘hook effect’. This occurrence is rare with the use of modern assays and may be reliably unmasked by repeating the PRL assessment after serum sample dilution [17, 22].

• Biotin is contained in many over-the-counter integrators and may cause artificially low PRL levels [23]. PRL measurement should be repeated after the withdrawal for a few days of these potentially interfering substances.

• Heterophilic antibodies are a rare cause of misdiagnosis. This unusual assay problem can be overcome with the appropriate treatment of the sample [24].

It is crucial to ask for sample dilution when the laboratory report does not indicate a precise PRL value but only states that the level is higher than for example 200 or 470 ng/mL (the levels corresponding to the upper limit of the calibration curve for the most widely employed immunoassay platforms). This information provides a well-defined baseline level for following up the treatment outcome.

After the diagnosis of true hyperprolactinemia, the extent of PRL increase generally correlates with the diagnosis. Levels higher than 250 ng/mL are most frequently observed in patients with MP, while a lower increase (up to 150 ng/mL) is usually detected in patients with microP or other causes of hyperprolactinemia [20], specifically:

• Physiological and para-physiological conditions: pregnancy, breast-feeding, and breast manipulation (piercing, mammoplasty).

• Stalk effect due to the disconnection between the hypothalamus and pituitary gland, with the consequent impairment of the inhibitory dopaminergic pathways. This condition may occur in large non-secreting adenomas and in tumoral, infectious, and inflammatory processes involving the hypothalamus, the perisellar region, the pituitary stalk and the pituitary, or in the primary empty sella.

• Non-endocrine diseases: liver cirrhosis, chronic renal disease, herpes zoster involving the chest wall, and neurinoma of intercostal nerves [9].

• Endocrine diseases: primary hypothyroidism [25] and polycystic ovary syndrome [9].

• Drugs that may interact with dopaminergic and/or serotoninergic regulation (Table **1**) [17, 26-28].

MRI examination of the hypothalamic-pituitary region should be performed with a standardized method to confirm that hyperprolactinemia is due to a pituitary lesion. T1- and T2-weighted sequences in the coronal and sagittal plane, without and with gadolinium, preferably with dynamic technique in case of microP, should be performed using at least 1.5 Tesla equipment. Pituitary adenomas are usually detected as mildly hypointense or isointense images on T1 and with a variable appearance on T2 sequences [29, 30]. Cystic and hemorrhagic components may also be present.

### 1.4 Treatment

Medical therapy with a dopamine agonist (DA) normalizes serum PRL levels in nearly 90% of patients with microP, and in 75–80% of patients with MP [31]. Importantly, tumor shrinkage is reported in more than 90% of treatment-naïve MP patients [32]. Cabergoline (Cab) and bromocriptine (Br) are the DA that are available in Italy. Both the 2006 guidelines of the Pituitary Society on the management of prolactinomas [33] and the 2022 Italian Position statement for clinical practice [34] state that Cab is the first choice DA due to its greater efficacy, tolerability, and sustained effect. In general, Cab is started at 0.25–0.5 mg weekly and given once or twice a week after dinner or at bedtime [35]. According to the clinical picture, the dose is uptitrated, if needed, at 1- to 3-month intervals in microP, and at weekly intervals in MP with visual impairment [36]. The maintenance dose is usually 0.5-2 mg/week and may be increased to 3.5 mg/week in a minority of MP.

Br is used only occasionally because it requires multiple daily administrations (2.5-10 mg/day fractionated in two or three doses) and is less well tolerated by the patients [37].

Adverse events induced by DA are usually transient and mild to moderate in severity: nausea, vomiting, postural hypotension, drowsiness, somnolence, nasal stuffiness, headache, Raynaud’s phenomenon, and constipation. The risk of apoplexy is low if the drug is started at a low dose. DA-induced neuropsychiatric symptoms are rare but may be worrisome. They include psychosis, or an exacerbation of pre-existing psychosis, and impulse control disorders (ICD), such as compulsive gambling, shopping, or eating, and hypersexuality [38, 39]. Current data do not support major concerns about the risk of valvopathy in hyperprolactinemic patients who are chronically treated with DA at standard doses (≤2 mg/week) [40, 41]. Subclinical valvular abnormalities detected by ultrasonography are not an indication for discontinuation of DA treatment. Finally, in patients with large invasive MP which erode the sellar floor, tumor shrinkage may cause cerebrospinal fluid (CSF) nasal leakage. Even if rarely reported, it requires urgent surgical repair [13].

In microP, treatment is aimed at restoring gonadal function (*i.e*., ovulatory menses in females, normal testosterone levels and sexual potency in males, and libido and fertility in both sexes) and at suppressing galactorrhea. Tumor shrinkage is not an issue in this setting because clinically significant or persistent growth is uncommon [34, 42]. Patients should be alerted for initial effective contraception because DA can quickly restore ovulation. Pregnancy should be programmed for timely DA discontinuation. DA should be withdrawn during pregnancy [34].

In MP, treatment is aimed mainly at the rapid relief of neuro-ophthalmologic symptoms and tumor shrinkage. In addition, the normalization of PRL levels with the restoration of eugonadism and fertility and suppression of galactorrhea should be pursued. Some patients experience an extremely rapid decrease in tumor size with a significant improvement in visual field defects within 24-72 hours. Cab achieves progressive PRL decrease and tumor shrinkage up to its disappearance or empty sella development, regardless of basal PRL levels or tumor size [34, 43]. DA dosage can often be safely tapered while keeping its efficacy.

DA resistance is defined as the failure to normalize PRL levels and to achieve at least 50% tumor size reduction on the maximally tolerated doses of DA [44]. In common clinical practice, the suggested maximum dose of Cab is around 4 mg per week [36]. At least 6 months of therapy on the highest tolerated DA dose is suggested as the minimum duration of treatment to define DA resistance [45]. The prevalence of resistant prolactinomas is estimated as high as 30% for Br and 10% for Cab treatment [46]. Resistance is more frequent in cases of MP and invasive tumors and in male patients [46-48]. In severe cases, with neuro-ophthalmologic impairment, patients should be tightly followed up at short intervals [34]:

• Neuro-ophthalmologic examination and clinical evaluation should be strictly scheduled during the first month of medical treatment to guide the choice of neurosurgical intervention in case of therapeutic failure.

• PRL levels should be assessed weekly or monthly during the first three months of therapy and subsequently at longer intervals if treatment is effective.

• MRI controls should be performed on the basis of the ophthalmologic and PRL changes induced by treatment.

In less severe cases of MP and in the absence of visual impairment, a less aggressive follow-up is appropriate, with the first evaluation after 3-6 months, and the following according to the clinical course: q 6-12 months in fully responsive patients and q 3-6 months in partially responsive subjects (achieving significant PRL decrease without normalization).

DA treatment discontinuation after long-term clinical and hormonal normalization (over two years) remains a partially unsettled issue [49]. Recent meta-analyses demonstrate that the remission rate, defined as the persistence of normal PRL values several months after drug withdrawal, is less than 40%, especially in MP [45, 46]. From a practical point of view, Cab treatment should not be withdrawn if PRL levels increase again after Cab dose tapering.

In case of withdrawal, PRL levels should be measured after three months and, thereafter, according to the results of this first control. MRI re-evaluation should be considered on the basis of the severity of PRL elevation.

In patients with MP remnant, the balance between the cost-effectiveness of simple yearly monitoring of PRL levels on minimal DA dosage and the intensive biochemical and neuroradiological monitoring aimed at withdrawing treatment should be individualized, also considering the impact of both strategies on quality of life (QoL).

Though DA often leads to hypogonadism reversal [50-52], some patients with MP require sex hormone replacement therapy for erectile dysfunction and gonadotropin treatment to restore fertility [53-57].

After the introduction of DA in the seventies of the last century, surgery was substantially abandoned and indicated as a second-line treatment in prolactinoma patients. Surgery was considered appropriate only in case of resistance or escape to DA, intolerance to DA, spontaneous or DA-induced CSF leakage, or for patients unwilling to undergo chronic treatment [58]. Due to the improvement in surgical techniques, transsphenoidal surgery may be now considered as a first-line option, given its high efficacy rate. A recent meta-analysis demonstrated that long-term disease remission is achieved after surgery in 74% of patients, regardless of surgical technique [59-62]. Specifically, long-term remission is reported in 83% of microP and in 60% of MP. Notably, the remission rate rises up to 89% for MP confined within the sella [63].

The rate of major complications is low (1-4%) [64, 65]: permanent diabetes insipidus, 0-5%; meningitis, 0-3%; and CSF leakage, 2-5%. Transient diabetes insipidus (7-28%), SIAD (5-14%), and hypopituitarism (1-4%) are also reported. On the other hand, improvement in pituitary function is observed in up to 35% of cases [64]. Post-surgical recurrence is described at 5 years in up to 18% of cases [66].

After surgery, PRL levels should be rapidly assessed (within a few hours), whereas MRI and a complete pituitary function evaluation should be postponed for 3-4 months [34].

Radiotherapy in patients with surgical failure or relapsing MP is aimed at control of tumor growth. Fractionated radiotherapy achieves tumor control in over 80% of cases and normalization of PRL levels in 20-30% of the patients [67, 68]. The technology most frequently employed is stereotactic radiosurgery with the use of gamma-knife or cyber-knife. It provides a focused high-energy beam of radiation to the biological target in a single fraction. A recent multicenter study involving the use of stereotactic radiosurgery showed tumor growth control in 95% of treated adenomas and normalization of PRL levels in 43% of the patients at 5 years and 54% at 8 years [69].

After radiation, a serial MRI and PRL monitoring is needed in most patients. An appropriate timetable of controls is every 3-6 months in the first year and subsequently, for several years, the schedule should be based on the initial response. When normalization of PRL levels is reached, the ongoing medical treatment with DA can be tapered, and withdrawn after persistent normalization of PRL levels. The pituitary function should also be serially monitored to rule out the risk of late hypopituitarism and to promptly start replacement treatment [67]. Hypopituitarism is reported to occur in 25% of irradiated patients [66].

As a general strategy, patients who are partially resistant to medical treatment may benefit from neurosurgery even if only an incomplete tumor resection may be achieved [41]. Surgical debulking may improve hormonal control and the post-operative dosage of Cab may be reduced [36, 62]. Patients who remain unresponsive to DA treatment after unsuccessful surgical treatment should be offered radiotherapy [46], while surgery may be repeated in resistant or aggressive cases.

In a minority of patients with complete resistance to DA treatment and unsuccessful surgery and radiation therapy, the tumor may relapse and show unrelenting and rapid growth [1, 68]. In these cases, chemotherapy should be considered. The only treatment approved in this setting is temozolomide, an alkylating agent, which has been demonstrated in a recent survey of the European Society of Endocrinology on 165 patients to induce a positive response, defined as a composite of complete, partial, or stable disease, in 79% of all patients [69]. Therapeutic options for patients with progression of disease during temozolomide treatment or recurrence of disease after an initial response are limited.

The aim of this guideline (GL) is to answer the clinical question: What is the best effective and safe treatment for PRL-secreting pituitary adenomas?

## 2 METHODS

This GL was developed according to the methodological manual for the production of clinical practice GLs developed by the National Center for the Clinical Excellence, Quality and Safety of Care of the Italian National Institute of Health (http://www.snlg-iss.it). Appendix **1** details the names and roles of all the people involved in the GL development team.

### 2.1 Clinical Question

The recommendations are the answers to a clinical question, formulated by the panel using the Population-Intervention-Comparison-Outcome (PICO) framework (Appendix **2**).

### 2.2 Selection of Outcomes

For each question, the panel identified potentially relevant outcomes, which were rated for their impact on therapeutic choices using a 9-point scale, namely:

• 1–3 points: outcomes of limited relevance

• 4–6 points: important, but not critical outcomes

• 7–9 points: critical outcomes.

Only outcomes classified as “critical” and “important” were considered in the systematic review of evidence and only those classified as “critical” were considered in the formulation of recommendations.

### 2.3 Literature Review and Assessment of the Quality of Evidence

A systematic search for each question was performed on the following databases: Cochrane Library, MEDLINE, Embase, Web of Science, and CINAHL (from inception to January 2021).

Specific search strategies were used for each database, as specified in each section of Appendix **3**. No time or language limits were imposed for all the searches. References of retrieved items were searched for further studies meeting inclusion criteria. A systematic review was performed through the following steps:

1. Selection of the eligible studies obtained with the initial search, based on title and abstract, for retrieval as full text.

2. Identification among retrieved full-text items of relevant studies, based on a priori inclusion and exclusion criteria.

3. Assessment of potential bias using validated instruments (AMSTAR 2) [70].

4. Extraction of main characteristics of the selected studies (enrolled population, considered outcomes, results), as summarized in tables.

5. Quantitative synthesis for each outcome by calculating odds ratio (OR) for categorical outcomes and weighted mean difference for continuous variables with 95% confidence intervals (CI). Quantitative meta-analysis was performed with RevMan 5.4 using fixed effects models.

6. Assessment of heterogeneity (I^2^) by the I^2^ statistic stating the percentage of variability in effects esteem due to heterogeneity rather than to chance.

7. The overall quality and strength of available evidence for outcomes selected by the panel were rated using the GRADE criteria.

8. Synthesis of results, using the GRADEPro Guideline Development tool (https://gradepro.org), with the frameworks EtD, which summarize results of systematic reviews for problem priority, desired and undesired effects of treatments, the strength of available evidence, values and preferences of stakeholders, economic resources needed, equity, acceptability, and feasibility of interventions.

### 2.4 Pharmacoeconomic Studies

The economic evaluation was performed by a pharmacoeconomist with specific expertise (MB).

A survey was performed among the GL panel members from different disciplines and regions that were representative of the Italian health system setting. The survey addressed the specific drivers that contribute to the total cost of each therapeutic procedure: cabergoline, bromocriptine, transsphenoidal surgery, with either microscopic or endoscopic technique, radiation treatment, or temozolomide. Specifically, for each procedure, we investigated the duration, type, and dosage of employed drugs, type and quantities of disposable materials, number and time of involvement of each operator, and percentage of patients requiring a caregiver during and after the procedure (indirect costs).

We calculated the mean value for each parameter to allow their use in the different regional settings under Italian National Health Service (NHS). Activity-based costing (ABC) analysis was utilized to estimate the expenditures associated with the provision of the different procedures [71]. ABC consists of three steps:

1. Resource identification by means of a specific survey among interdisciplinary panelists. The resources required to implement the procedures under investigation were detailed to quantify each component (time of operators’ activities, materials, drug dosage, technical resources, *etc*.).

2. Cost measurement by consultation of scientific literature and specific databases (such as price lists) [72-79].

3. Results’ valorization: the data obtained during the previous steps were combined to define the full cost of each action and the whole process [80].

The GL economic analysis evaluated the four large resource categories employed in the procedure under investigation:

• Direct cost paid by NHS for drugs.

• Direct cost paid by NHS for disposable materials.

• Direct cost paid by NHS for the working time of operators and the use of structures.

• Indirect costs sustained by caregivers.

To assess the costs driven by the complications of treatments, we evaluated the rate of occurrence for each potential complication induced by the various procedures, and the generated costs were expressed as the corresponding fraction. Namely, if the cost of a specific complication was € 5000, including all the drivers (employed drugs, hospital stay, and loss of productivity), and if the complication is reported to occur in about 1% of patients, the sum of € 50 was added to the total cost of the procedure under evaluation.

### 2.5 Cost-efficacy Analysis

A cost-efficacy analysis (CEA) was carried out to provide information on the economic sustainability of the considered treatments for the management of patients with microP in the Italian healthcare setting.

CEA is a widely used tool for evaluating public policies, particularly in the health sector. In pharmacoeconomics, the incremental cost-effectiveness ratio (ICER) of a therapeutic or preventive intervention is the ratio between the incremental costs and the incremental outcome given by the comparison between the interventions under assessment. The selection of the appropriate outcome measure should be based on clinical judgment in the context of the intervention under evaluation.

Cost-utility analysis is a CEA where the outcome measure is expressed as quality-adjusted life years (QALYs). A QALY is generated when the patient experiences one year in a state of full health. If the patient experiences a state of not complete health during the same time frame, the QALY generated by this action is considered lower than one. For the evaluation of the cost-utility profile of a therapeutic alternative in the Italian context, as compared to the strategies already available in the setting, reference is made to a willingness to pay (WTP) threshold to gain an additional QALY, set to assess the acceptability of the new strategy, should this be associated to both higher QALYs and higher costs. As regards the Italian setting, the WTP threshold is set to approximately € 40,000/QALY gained [81]. Treatments whose cost-utility profile exceeds such index are deemed not economically sustainable by the reference economy.

### 2.6 Development of Recommendations

The GL panel examined and discussed each clinical question: the EtD frameworks, the tables of evidence, and the summaries of results (forest plots of meta-analyses). The GL panel formulated recommendations, rated either as strong or weak, based on the priority of the problems, benefits and harms of the options, the strength of evidence, values and preferences, use of resources, feasibility, acceptability, and equity of the procedure. Disagreements were settled through collective discussion in all cases. If evidence was not available or it was inappropriate for a formal rating of the quality of evidence, the GL panel developed indications for good clinical practice to be used as instructions complementary to recommendations.

### 2.7 External Review

The panel appointed an interdisciplinary board of external reviewers with specific expertise in pituitary disease management. External reviewers received the draft version of the GL and submitted their comments to the panel, which included, after a dedicated discussion, the amendments to the GL document.

### 2.8 Value of Recommendations

Quality of evidence was rated as:

• High: highly reliable data whose confidence in estimated effects is unlikely to be modified by further studies.

• Moderate: moderately reliable data whose confidence in estimated effects could be modified by further studies.

• Low: still limited and uncertain results that need further research for a reliable assessment of the positive and negative effects of the intervention.

• Very low: available data that are not reliable and the estimates of effects should be considered with caution.

The strength of recommendations was rated as strong or weak.

A strong recommendation implies the following:

• For clinicians: the majority of patients should receive the recommended intervention.

• For patients: almost all properly informed patients should follow the recommendation, whereas only a small fraction of them may choose different options.

• For policymakers: the recommendation can be employed for planning the use of the available resources.

A weak recommendation implies the following:

• For clinicians: the final choice should include careful consideration of patients’ values and preferences.

• For patients: the majority of properly informed patients will follow the recommendation, but a minority of them may choose different options.

• For policymakers: a discussion involving the stakeholders should be performed on the issue.

## 3 RESULTS

The PRISMA flow diagram for the selection of the studies is illustrated in Appendix **4**. Seven systematic reviews were retrieved [40, 56, 82-86]. The methodological quality evaluation of the selected studies is detailed in Appendix **5**.

### 3.1 Cabergoline *Versus* Different PRL-suppressing Drugs or no Treatment

Two systematic reviews were included. The first considered the randomized controlled trials (RCTs) that compared Cab and Br in patients with prolactinoma or idiopathic hyperprolactinemia [82]. The second included cross-sectional studies that compared Cab with no treatment [85]. The methodological quality was critically low. To overcome the lack of data on different comparisons and outcomes, we also identified reviews that included studies without a control group and that, in some cases, considered a population presenting various causes of hyperprolactinemia.

A systematic review of case series (104 patients with giant prolactinoma, 77 men and 27 women) evaluated the efficacy of DA treatment for 42.63 months (mean, range 6-204): Br 7.5-15 mg/day in 55 patients and Cab 1-1.5 mg/week in 53 patients, without a control group [86]. Tumor shrinkage and improvement of visual field defect were not statistically different with the use of the two drugs. PRL normalization was more frequent on Cab (60.4% *vs*. 35.3%), but the difference occurred only in males.

A systematic review of observational studies reported pituitary apoplexy in 16 out of 157 patients treated with DA for a period of 3-15 months, with a wide range of incidence (1.19-44.83% of cases) [84]. Only one of the included studies reported that DA treatment could be associated with a protective role against apoplexy.

A systematic review [56] of 55 observational studies without control group, including 3564 patients (median age 41 years, 70% women), reported disease remission after DA treatment for 24 months (mean, range 1-162) and drug discontinuation for 12 months (mean, range 2-90) in 32% (95% CI 18-48%) of cases (microP 52%, 95% CI 44-59%; MP 28%, 95% CI 4-61%). PRL levels were normalized on treatment in 88% of the whole group (95% CI 82-94%), in 90% of microP (95% CI 84-95%), in 86% of MP (95% CI 77-93%), and in 42% of giant adenoma (95% CI 23-62%). Frequent side effects were fatigue in 30% (95% CI 19-42%), libido alterations in 28% (95% CI 22-36%), sleep disturbances in 25% (95% CI 17-34%), and nausea in 17% (95% CI 2-41%). Major adverse events were ICD in 3% (95% CI 1-6%), compulsive gambling in 6% (95% CI 3-11%), and CSF leakage in 4% (95% CI 1-10%).

A systematic review of 13 case-control studies (836 patients with hyperprolactinemia on Cab treatment for 37-80 months, with a cumulative Cab dosage of 173-443 mg, *vs*. 1388 controls) demonstrated that risk of tricuspid insufficiency, but not of aortic or mitral lesions, was increased on Cab treatment when protracted over 12 months (OR, 3.74; 95% CI 1.79-7.80; *p* < 0.001) [40].

### 3.2 Transsphenoidal Surgery

A systematic review [56] of 25 observational studies without a control group involving 1836 patients (median age 34 years, 69% women) reported disease remission after a median follow-up of 22 months (range 3-135) in 67% of cases (95% CI 60-74%). Specifically, remission was observed in 83% of microP (95% CI 76-90%) and in 60% of MP (95% CI 50-70%) with no reported mortality (95% CI 0-1%). Major complications were the occurrence of persistent diabetes insipidus in 2% (95% CI 0-55), meningitis in 1% (95% CI 0-3%), and CSF leakage in 3% (95% CI 2-5%) of cases. Further endocrine complications were transient diabetes insipidus in 16% (95% CI 7-28%), SIAD in 9% (95% CI 5-14%), and hypopituitarism in 2% (95% CI 1-4%).

### 3.3 Temozolomide Treatment

A systematic review of case series and case reports described the results of oral temozolomide (150-200 mg/m^2^ for 5 days to 28 days, for 1 to 24 cycles) in patients with aggressive or resistant prolactinoma (23 studies) or PRL-secreting carcinoma (19 studies) [83]. All included patients were resistant to standard treatments (DA, somatostatin analogs, repeated surgery, radiation) with tumor progression. On temozolomide therapy, tumor shrank in 25/34 patients (73.5%), and PRL was significantly reduced in 18/24 patients (75%) and normalized in 2/24 patients (8.3%). Tumor progression was observed in 7/34 patients (20.6%). Most patients tolerated the treatment well. Severe adverse events, mostly hematological toxicity, were reported in three cases and required discontinuation of therapy in one patient.

### 3.4 Economic Evaluation

Tables **2** and **3** show the weighted costs of the therapeutic alternative strategies for the management of micro and macroprolactinomas, respectively.

### 3.5 Cost-efficacy Analysis

The analysis adapted the results of the study by Jethwa *et al*. [87] in the American context to the Italian healthcare setting. In that study, the pharmacological treatment based on cabergoline and bromocriptine was compared with the use of endoscopic and microscopic surgery. To determine the cost-effectiveness of surgical management compared to the use of pharmacological alternatives, a maximum threshold of WTP equal to € 40,000/QALY gained was considered.

A probabilistic tree, based on the natural history of the pathology, was developed for the realization of the analysis. The study also included the realization of a multivariate probabilistic analysis, performed to characterize the uncertainty surrounding the parameters considered in the pharmacoeconomic model: 1000 simulations were performed considering a deviation in the absolute value of each parameter in its range of variation. In the comparison of the average cost and utility values of the pharmacologic and surgical alternatives, it appears that surgery is a cost-effective alternative with an ICER of € 35,248.13 (Table **4**).

### 3.6 Recommendations

The following recommendations were issued by the GL panel, based on the reported analyses, for the clinical question “What is the best effective and safe treatment for PRL-secreting pituitary adenomas?”

**Recommendation 1**: The panel recommends the pharmacologic treatment with cabergoline as the first-line therapy vs. the alternative dopaminergic drug bromocriptine. Cabergoline should be employed at the lowest effective dose capable of controlling the clinical picture (strong recommendation, very low quality of evidence).

• **Evidence**: Though only limited and low-quality evidence has compared the outcomes of the two drugs, indirect evidence stands in favor of Cab. A systematic review of observational studies without a control group [56] points to a modest benefit for therapeutic efficacy of Cab *vs*. Br associated with robust benefit for tolerability. The panel believes that Cab acceptability is higher for the patients due to its schedule (administration once or twice per week as compared to Br that requires at least daily administration). Though this dimension was not formally evaluated in an ad hoc study, the obviously superior acceptability together with better efficacy and tolerability induced the panel to upgrade the strength of the recommendation, notwithstanding very low-quality evidence.

Subgroup Indications:

• **In patients with microP:** we recommend as the aim of treatment the reversal of the clinical condition with a main focus on hypogonadism.

• **In patients with MP on chronic DA treatment with persisting tumoral tissue and pathologic PRL levels:** We recommend against DA withdrawal.

• **In women with microP after menopause:** We recommend DA withdrawal.

• **In women with MP after menopause:** We recommend continuing DA treatment at the lowest effective dose capable of controlling tumor growth, with a follow-up planned on the basis of clinical status.

• **Evidence**: Though women after menopause were not specifically considered in the available evidence, the panel addressed the issue of the duration of drug treatment. Literature data demonstrated that treatment can be safely discontinued in postmenopausal women with microP, whereas a more prudent attitude is warranted in other conditions [34].

• **Indications for further research:** RCT with an adequate sample size comparing the efficacy and safety of Cab *vs*. Br should be carried out.

**Recommendation 2:** The panel suggests that cabergoline and trans-sphenoidal resection of the adenoma should be offered as alternative therapeutic options to any patient with a fully resectable adenoma (microP or enclosed MP). The dialogue with the patient should be preferably conducted during a joint evaluation among pituitary experts. This approach allows the patient to select the option that he/she considers more appropriate according to his/her general conditions, values, preferences and accessible resources (weak recommendation, very low quality of evidence).

**Evidence:** A cohort study showed that a few patients with prolactinoma opted for neurosurgery instead of long-term medical therapy [88]. The surgical option appears also preferable from an economic point of view.

**Recommendation 3:** The panel suggests bromocriptine treatment in patients with intolerance to cabergoline who are not candidates for surgery (weak recommendation, very low quality of evidence).

**Recommendation 4:** The panel recommends the resection of the adenoma by an expert pituitary surgeon for patients:

• Who do not exhibit rapid improvement of neuro-ophthalmologic impairment after two weeks of cabergoline treatment.

• Who are resistant/intolerant to cabergoline or other DA.

• Who escape from DA effects.

• Who require treatment but are unwilling to take chronic medical therapy (strong recommendation, very low quality of evidence).

**Evidence**: Although the quality of the available evidence on surgical therapy is low, overall data from the literature point to the benefits of this therapy, especially in some subgroups of patients. A systematic review of observational studies without a control group [56] confirmed the efficacy of surgery compared to pharmacological therapy. In patients who do not benefit or tolerate drug therapy or who refuse chronic medical treatment, neurosurgery is the only therapeutic option with adequate safety and demonstrated efficacy. Thus, the GL panel increased the strength of the recommendation notwithstanding very low-quality evidence.

**Recommendation 5:** The panel recommends, in case of uncontrolled tumor growth despite treatment with DA associated with neurosurgery, that a multidisciplinary pituitary team with specific expertise adopts the multimodal approach that is appropriate for the individual patient. The use of repeated surgery + radiotherapy + DA + temozolomide should be considered with a time frame appropriate to the course of the disease (strong recommendation, low quality of evidence).

**Evidence:** Patients who show tumor progression despite the appropriate use of standard therapies are at risk of unfavorable evolution and of death if are not promptly addressed to a multi-disciplinary team, including different skilled experts. Notwithstanding low-quality evidence, the panel increased the strength of the recommendation for rare patients with potentially life-threatening clinical conditions.

### 3.7 Indications for Good Clinical Practice

The following statements reflect the opinions of the GL panel members about issues not addressed by studies directly comparing the different therapeutic options. These statements are complementary to the formal recommendations, are based on large clinical experience, and are unanimously agreed upon by the panel. Thus, they are provided as an aid for good clinical practice.

1. Whenever neurosurgery may be considered, the patient should be referred to a pituitary surgeon with specific expertise.

2. Clinical surveillance may be considered an appropriate option in patients with microP without hypogonadism or galactorrhea.

3. For patients, especially those of the male sex, who start Cab treatment, their caregivers should be warned about the possible development of impulse control disorders. They should be regularly investigated for the occurrence of psychiatric symptoms during chronic treatment. These adverse effects occur rarely but may be severe [89], and clinicians should be aware of their possibility.

4. After DA initiation, MRI control should be performed within 12 months in patients with microP and within 3-6 months in those with MP. Earlier follow-up should be considered in non-responder MP subjects and in case of new symptoms occurrence. The use of contrast media during follow-up should be appropriately limited, especially in patients with MP, to avoid harm related to Gd deposits.

5. In patients with MP who are DA-responders, follow-up can be safely performed after PRL normalization and tumor shrinkage with PRL assessment performed at yearly intervals.

6. Urgent evaluation by a neurosurgeon is warranted in case of CSF leakage on DA treatment.

7. In patients on protracted cabergoline treatment, especially in those taking high doses (*e.g*., >2 mg/week for prolonged periods), cardiac ultrasonography should be considered on the basis of a complete clinical evaluation, including age, comorbidities, Cab dosage, and duration of treatment. Potential extra-endocrine causes of valvular involvement should be ruled out. The decision to undergo ultrasonography screening needs an individualized comprehensive assessment considering all the potential additional risk factors.

8. In patients with MP, DA treatment may be discontinued only after the complete disappearance of the tumor mass, or an at least 50% decrease in tumor size, associated with the persistence of low-normal PRL levels after progressive down-titration of DA. In these patients, a careful quarterly follow-up of PRL levels and gonadal status should be performed.

9. In males with MP and persistent hypogonadism, testosterone replacement treatment should be started within 3-6 months after the start of DA treatment, provided that PRL is progressively decreasing. A laboratory re-evaluation of the gonadal axis should be performed after the normalization of PRL levels.

10. In hypogonadal women with MP, sex hormone replacement therapy should be based on a case-by-case evaluation.

11. Hormone replacement treatment in hypogonadal women with MP should be continued at least until the age of physiologic menopause.

12. Women with microP or DA-responder MP can be safely treated with estroprogestinic if they require contraception.

13. Hormone replacement treatment can be offered to postmenopausal women with microP, provided that PRL levels are monitored.

14. Patients with MP should be tightly followed up with the determination of PRL levels and MRI assessment. This approach is of relevant importance in males who are diagnosed after the age of 50 years due to their elevated risk of unfavorable course.

15. Whenever radiation treatment is indicated, stereotactic radiosurgery should be preferentially used instead of fractionated radiotherapy, unless the tumor is huge or close to the optic pathways.

16. In patients with invasive MP or pituitary carcinoma who are unsuccessfully operated on and/or irradiated due to uncontrolled tumor growth, temozolomide therapy should be started under the guidance of a neuro-oncologist.

### 3.8 Guideline Update

This systematic review will be updated with the use of the same search strings, three years from the date of GL approval. The ERT and the GL panel will assess the availability of new clinical data that could modify the overall quality of evidence and risk/benefit ratio and, consequently, the formulation and strength of recommendations.

The GL panel will also consider updating, adding, or removing clinical questions or outcomes of interest and their relative relevance. In case of changes in clinical questions and/or critical outcomes, the process of evidence review and development of recommendations will be performed again.

## 4 DISCUSSION

Pharmacological therapy is the standard of care for prolactinomas since the introduction, in the late 70s of the last century, of DA drugs with proven anti-secretory and anti-tumor efficacy. DA drugs have been used successfully in the majority of cases, while neurosurgery has been progressively reserved for a minority of patients, namely those intolerant or resistant to pharmacological therapy and those with aggressive tumors [7].

In recent years, the technical progress in surgical techniques has resulted in a significant improvement in neurosurgical outcomes, paralleled by a decrease in adverse events. Currently, neurosurgery is considered also for non-aggressive prolactinomas, with an extension of the indications to micro-adenomas and to enclosed macro-adenomas, in the event of patients who are unwilling to undergo long-term drug therapy [55-57]. Besides being appreciated by part of the patients [88], surgical treatment is considered a favorable cost-effective approach [87].

The above-reported analysis assessed, at the best accuracy level, the costs associated with pharmacological and surgical interventions for patients affected by microP or MP. The results of a survey addressed to a pool of clinicians with proven clinical experience within the Italian healthcare setting were used for this issue. The survey investigated drugs, tests, visits, and health professionals involved in the treatment process and the contribution provided by the caregiver/family members. The analysis showed that the average absorption of resources per patient with microP and MP is in the first year, respectively.

• Cabergoline, € 1,586.04 and € 2,267.46.

• Bromocriptine, € 1,129.09 and € 1,636.21.

• Temozolomide, not appropriate, and € 7,738.80.

• Endoscopic surgery, € 8,818.75 and € 8,897.45.

• Microscopic surgery, € 8,506.63 and € 8,512.11.

• Radiation therapy, not appropriate, and € 3,214.06.

As for the loss of patients and caregivers’ productivity, costs are € 256.59 for pharmacologic therapies, € 1,399.95 and € 924.54, respectively, for endoscopic and microscopic surgery, and € 788.70 for irradiation. Notably, the costs of health personnel for surgical treatment could be considered less relevant due to the use of already available resources. Actually, the National Health Service staff is paid regardless of whether or not the service under evaluation is provided.

The cost-utility analysis performed by Jethwa *et al.* [87] on the subgroup of patients with microP could be modified according to the Italian healthcare setting. This investigation demonstrated that cabergoline is a more cost-effective treatment than bromocriptine, with an ICER of € 3,171.48/QALY. Also, endoscopic surgery was more cost-effective than microscopic surgery, resulting in an ICER of € 21,242.20. Finally, surgical management, regardless of its technical modalities, was more cost-effective than the pharmacologic approaches, with an ICER of € 35,248.13. A major limitation in the determination of the management costs of prolactinoma is the poor quality of available evidence concerning the therapies under analysis in the Italian context.

The large-scale implementation of a shift from DA as the first-line approach for prolactinomas deserves a few comments. The favorable results reported in the literature are obtained in centers of excellence, which are not easily accessible from all areas of the country. A pituitary neurosurgeon with specific expertise is requested to perform at least 50 pituitary surgeries per year and work in a multi-disciplinary pituitary team [90]. The working group should include an endocrinologist and a neuroradiologist, but the participation of neuro-ophthalmologist, neuro-oncologist, radiotherapist, and pathologist is advisable.

Based on this consideration, two management scenarios can be foreseen in the medium term. Patients may be addressed to centers with lower experience, at the cost of obtaining suboptimal results or, alternatively, to centers of excellence, so resulting in delayed admission times and interventions for pituitary diseases that need a rapid action, such as in the event of severe hypercortisolism due to ACTH-secreting adenomas and tumors abutting optical pathways.

Unfortunately, it was not possible to have an accurate “official” report of transsphenoidal surgeries that are performed each year in Italy, because the diagnosis-related group (DRG) 286 aggregates the surgeries on the adrenal and pituitary glands (3190 in 2019 according to the 2020 annual report of the Ministry of Health). Presently, 5-10% of pituitary surgeries are performed for prolactinomas. The number of operations performed with curative intent is widely variable between Italian centers but it can be arbitrarily assumed that nearly 90% of prolactinomas are operated upon for optical pathway compression, for aggressive growth, or for resistance to medical therapy. We postulate that the implementation of this GL might result in an increase in the annual number of surgeries performed as first-line treatment for prolactinomas. The estimated raw cost for the Italian NHS could initially range from € 1,600 to € 12,000 per year. Thus, if the proportion of prolactinoma patients undergoing neurosurgery as first-line treatment would increase by 10% yearly (a conservative and absolutely arbitrary fraction), the NHS excess cost could rise to € 7,000-32,000 in a three-year period. Though a conclusive estimate of the variation of annual expenses cannot be performed, the cost-efficacy analysis appears in favor of surgical therapies. The postulated ICER of € 36,122 for surgical therapies compared to pharmacologic treatment appears well below the assumed expenditure threshold for each QALY, which is set at € 40,000 in our economic setting.

Major limitations to a reliable calculation of costs changes are as follows:

• Price fluctuation of surgical devices.

• Risk of surgical complications, and related costs, which is most likely higher in the real world than in series reported by specialized centers.

• Overestimation of costs for surgical procedures due to a postulated similar follow-up for pharmacologic treatment and neurosurgery. As the recurrence rate of prolactinomas in post-surgical remission is low (25% at 10 years) [91], follow-up after surgery could be less intense than with pharmacologic therapy.

• Costs of replacement therapies and monitoring that may result from surgery-induced hypopituitarism.

• Personnel cost for surgical interventions also including the pauses between operations and the non-surgical times (dressing and undressing times, patient information, informed consent, operating room cleaning, monitoring of patient weaning from anesthesia, *etc*.).

## CONCLUSION

In conclusion, based on GRADE methodology, this is the first GL that considers pharmacologic and surgical options as first-line treatments of equivalent importance for PRL-secreting enclosed adenomas. This innovative approach should not be considered as backward movement of a swinging pendulum but as a forward step that may enable clinicians and patients to consider the best management for this frequent disease with the potential of a definitive cure.

## Figures and Tables

**Table 1:** Common PRL-rising medications.

Anti- Psychotic Drugs	First-generation or Typical Anti-psychotics: Phenothiazines, Thioxanthenes, Butyrophenones Second-generation Atypical Neuroleptic Drugs: Amisulpiride, Risperidone
Anti-depressant drugs	Tricyclic: imipramine, amitriptyline Selective serotonin reuptake inhibitors
Cardiovascular drugs	Reserpine, verapamil, α-methyl-DOPA
Gastrointestinal drugs	Metoclopramide, domperidone, L-sulpiride, cimetidine, ranitidine
Miscellany	Opioids, morphine, cocaine, marijuana Anesthetics Estrogens

**Table 2:** Costs of therapeutic procedures – microprolactinoma.

**Procedure**	**Application**	**1^st^ year**	**Following Years**	
			**(2^nd^-5^th^)**	**(Beyond 5^th^)**
Cabergoline	91.44%	€ 1,586.04	€ 807.32	€ 765.59
Bromocriptine	1.71%	€ 1,129.09	€ 466.69	€ 438.91
Temozolomide	0%	N/A	N/A	N/A
Endoscopic surgery	10.63%	€ 8,818.75	€ 319.91	€ 294.64
Microscopic surgery	0.86%	€ 8,537.54	€ 324.64	€ 316.96
Radiation	0%	N/A	N/A	N/A
**Weighted Total**		**€ 2,558.91**	**€ 798.13**	**€ 754.85**

**Table 3:** Costs of therapeutic procedures – macroprolactinoma.

**Procedure**	**Application**	**1^st^ Year**	**Following Years**	
			**(2^nd^-5^th^)**	**(Beyond 5^th^)**
Cabergoline	91.44%	€ 2,267.46	€ 1,078.26	€ 967.74
Bromocriptine	1.71%	€ 1,636.21	€ 737.00	€ 607.26
Temozolomide	1.14%	€ 7,738.80	€ 1,052.34	€ 1,014.07
Endoscopic surgery	10.63%	€ 8,897.45	€ 517.73	€ 422.93
Microscopic surgery	0.86%	€ 8,512.11	€ 446.97	€ 384.52
Radiation	2.46%	€ 3,214.06	€ 615.72	€ 538.76
**Weighted Total**		**€ 3,287.53**	**€ 1,084.59**	**€ 968.37**

**Table 4:** Results of cost-efficacy analysis for therapeutic actions.

-	QALYs	Costs	Differentials		ICER
			**Costs**	**QALYs**	
Microscopic surgery	0.9656	€ 8,613.44	-	-	-
Endoscopic surgery	0.9725	€ 8,759.12	€ 146.31	0.01	€ 21,242.20
Mean for surgery	**0.9691**	**€ 8,686.60**	**-**	**-**	**-**
Bromocriptine	0.7939	€ 3,616.20	-	-	-
Cabergoline	0.8628	€ 3,834.61	€ 218.40	0.07	€ 3,171.48
Mean Pharmacologic Treatments	**0.8283**	**€ 3,725.40**	**€ 4,961.20**	**0.14**	**€ 35,248.13**

## LIST OF ABBREVIATIONS

ABC: Activity-based costing
ACTH: Adrenocorticotropic hormone
AGREE: Appraisal of guidelines for research and evaluation
AME: Associazione medici endocrinologi
AMSTAR: A measurement tool to assess systematic reviews
Br: Bromocriptine
Cab: Cabergoline
CEA: Cost-efficacy analysis
CI: Confidence interval
CINHAL: Cumulative index to nursing and allied health literature
CSF: Cerebrospinal fluid
DA: Dopamine agonist
DOPA: Dihydroxy-phenylalanine
DRG: Diagnosis-related group
ERT: Evidence review team
EtD: Evidence to decision
FIPA: Familial isolated pituitary adenoma
GH: Growth hormone
GL: Guideline
GRADE: Grading of recommendations assessment, development, and evaluation
ICER: Incremental cost-effectiveness ratio
IRCCS: Istituto di ricovero e cura a carattere scientifico
IRP: International reference preparation
MEN: Multiple endocrine neoplasia
microP: Micro-prolactinoma
MP: Macro-prolactinoma
MRI: Magnetic resonance imaging
N/A: Not appropriate
NHS: National health service
OR: Odds ratio
PICO: Population, intervention, comparison, outcome
PRL: Prolactin
QALY: Quality-adjusted life years
QoL: Quality of life
RCT: Randomized controlled trial
SIAD: Syndrome of inappropriate anti-diuresis
WHO: World Health Organization
WTP: Willingness-to-pay

## Appendix 1

### GUIDELINE DEVELOPMENT TEAM

**Chair**: Renato Cozzi (endocrinologist, ASST Grande Ospedale Metropolitano Niguarda, Endocrinology Department, Milan).

### PANEL MEMBERS:

• Renata Simona Auriemma (endocrinologist, Departments of Molecular and Clinical Endocrinology and Oncology ‘Federico II’ University of Naples).

• Ernesto De Menis (endocrinologist, Internal Medicine 2, Treviso Hospital, Treviso; Functional Department of Endocrinology and Metabolism, AULSS 2 Veneto).

• Felice Esposito (neurosurgeon, Division of Neurosurgery, University of Messina).

• Emanuele Ferrante (endocrinologist, Endocrinology Unit, Fondazione IRCCS Ca’ Granda Ospedale Maggiore Policlinico, Milan)

• Giuseppe Iatì (radiotherapist, Department of Radiation Oncology, University of Messina).

• Diego Mazzatenta (neurosurgeon, Department of Biomedical and NeuroMotor Sciences (DIBINEM), University of Bologna, IRCCS Istituto delle Scienze Neurologiche di Bologna, Programma Neurochirurgia Ipofisi - Pituitary Unit, Bologna).

• Maurizio Poggi (endocrinologist, Endocrinology, Department of Clinical and Molecular Medicine, S. Andrea Hospital, Sapienza University of Rome).

• Roberta Rudà (neuro-oncologist, Division of Neurology, Castelfranco Veneto and Treviso Hospital, Treviso; Division of Neuro-Oncology, Department of Neuroscience “Rita Levi Montalcini”, University of Turin).

• Fabio Tortora (neuroradiologist, Radiology Unit, Department of Advanced Biomedical Sciences, University “Federico II”, Naples).

### EVIDENCE REVIEW TEAM:

• Fabio Cruciani, Zuzana Mitrova, Rosella Saulle, Simona Vecchi (Department of Epidemiology, Regional Health Service - ASL Roma1, Regione Lazio).

• Michele Basile (High School of Economy and Management of Health Systems, Catholic University of Sacred Heart, Rome).

### EXTERNAL REVIEWERS:

• Alessandro Bozzao (neuroradiologist, Neuroradiology, S. Andrea Hospital, NESMOS Department - Neuroscience, Mental Health, Sensorial Organs -, Sapienza University of Rome).

• Marco Caputo (laboratory physician, Laboratorio Analisi Cliniche e Microbiologia, Synlab SRL, Calenzano, FI).

• Francesco Doglietto (neurosurgeon, Institute of Neurosurgery, Catholic University School of Medicine, Rome).

• Francesco Ferraù (endocrinologist, Medical Oncology Department, San Vincenzo Hospital, Taormina, ME).

• Andrea Gerardo Lania (endocrinologist, Department of Biomedical Sciences, Endocrinology Unit, Humanitas Clinical and Research Center IRCCS, Humanitas University, Rozzano, MI).

• Stefano Laureti (endocrinologist and general practitioner, USL Umbria 1, Perugia).

• Stefano Lello (gynecologist, Department of Woman and Child Health and Public Health, Institute of Obstetrics and Gynecology, Fondazione Policlinico Universitario Agostino Gemelli IRCCS, Rome).

• Davide Locatelli (neurosurgeon, Division of Neurosurgery, Department of Biotechnology and Life Sciences, University of Insubria, ASST Sette Laghi, Varese).

• Pietro Maffei (endocrinologist, Department of Medicine, 3^rd^ Medical Clinic, Padua University).

• Giuseppe Minniti (radiotherapist, Department of Medicine, Surgery and Neurosciences, University of Siena).

• Alessandro Peri (endocrinologist, Pituitary Diseases and Sodium Alterations Unit, AOU Careggi, Department of Experimental and Clinical Biomedical Sciences “Mario Serio”, University of Florence).

• Chiara Ruini (psychologist, Department of Psychology, University of Bologna).

• Fabio Settanni (laboratory physician, Clinical Biochemistry Laboratory, City of Health and Science University Hospital, Turin).

• Antonio Silvani (neuro-oncologist, Department of Neuro-Oncology, Fondazione IRCCS Istituto Neurologico Carlo Besta, Milano).

• Nadia Veronese (nurse, Poliambulatorio Medico, DH/MAC Endocrinology, Pneumology, Diabetology, Allergology, ASST Grande Ospedale Metropolitano Niguarda, Milan).

### AME GUIDELINE TEAM

• Alessandro Scoppola, team coordinator (endocrinologist, Endocrinology Unit, Ospedale Santo Spirito, Rome)

• Roberto Attanasio (endocrinologist, AME Scientific Committee, Milan)

• Agostino Paoletta (endocrinologist, ULSS6 Euganea Endocrinology, Padova)

• Enrico Papini (endocrinologist, Endocrinology & Metabolism Department, Ospedale Regina Apostolorum, Albano Laziale, Rome)

• Agnese Persichetti (endocrinologist, Ministry of the Interior, Rome)

• Irene Samperi (endocrinologist, Endocrinology Unit, ASL Novara)

## Appendix 2

### CLINICAL QUESTION AND INCLUSION CRITERIA (PICO)

**Clinical question:** What is the best effective and safe treatment for PRL-secreting pituitary adenomas?

**Population**: Adult subjects (aged ≥18 years) diagnosed with PRL-secreting pituitary adenoma.

**Intervention:** Chronic dopaminergic treatment with cabergoline.

### COMPARISONS:

a) Other pharmacologic treatments (bromocriptine, methergoline).

b) Neurosurgery.

c) Radiation treatment (fractionated or radiosurgery).

d) Temozolomide.

e) No intervention (surveillance).

### OUTCOMES:

1. Permanent cure allowing treatment withdrawal.

2. Tumor shrinkage or disappearance with the improvement of local signs and symptoms.

3. PRL reduction/normalization.

4. Treatment resistance or escape.

5. Improvement or cure of hypogonadism and its complications; recovery of pituitary function and related complications (hypothyroidism, adrenal insufficiency, diabetes insipidus).

6. Complications of treatments.

7. Quality of life.

**Design of searched studies:** Systematic reviews and metanalysis of RCTs or observational studies were included. If unavailable, individual RCTs or observational studies with a control group were searched. If different relevant reviews were retrieved for the same clinical question, the review with the higher methodological quality, according to the checklist AMSTAR 2, was selected.

Subgroups of patients with microprolactinoma, macroprolactinoma, or giant prolactinoma were considered.

**Exclusion criteria:** Pregnant women, patients with plurihormonal secreting adenomas or familiar/genetic forms.

## Appendix 3

### DATABASE: CENTRAL

#### DATE OF SEARCH: ISSUE 1, 2021

1. MeSH descriptor: [Prolactinoma] explode all trees

2. Prolactinoma*

3. Microprolactinoma*

4. Macroprolactinoma*

5. MeSH descriptor

6. Prolactin near/2 secreting

7. PRL near/2 secreting

8. prolactin*:ti

9. {OR #1-#9}

10. MeSH descriptor: [Dopamine Agonists] this term only

11. dopamin* NEXT agonist*

12. cabergolin*

13. 11 OR #12 OR #13

14. 10 AND #14 in Trials

### DATABASE: OVID MEDLINE(R)

#### DATE OF SEARCH: 1946 TO JANUARY 7, 2021

1. Prolactinoma/

2. Prolactinoma*.mp.

3. Micro?prolactinoma*.mp.

4. Macro?prolactinoma*.mp.

5. (prolactin* adj3 (adenoma* or carcinoma* or tumor*)).tw.

6. Lactotroph.mp. or Lactotrophs/

7. PRL.ti.

8. (PRL secreting adj3 (adenoma* or carcinoma* or tumor*)).tw.

9. prolactin*.ti.

10. Dopamine Agonists/

11. dopamin* agonist*.ti.

12. cabergolin*.mp. or Cabergoline/

13. 10 or 11 or 12

14. 1 or 2 or 3 or 4 or 5 or 6 or 7 or 8 or 9

15. 13 and 14

### DATABASE: EMBASE

#### DATE OF SEARCH: 1974 TO JANUARY 7, 2021

1. Prolactinoma/

2. Prolactinoma*.mp.

3. Micro?prolactinoma*.mp.

4. Macro?prolactinoma*.mp.

5. prolactin*.ti.

6. Lactotroph.mp. or prolactin secreting cell/

7. PRL.ti.

8. (PRL secreting adj3 (adenoma* or carcinoma* or tumor*)).tw.

9. (Prolactin* adj3 (adenoma* or carcinoma* or tumor*)).tw.

10. 1 or 2 or 3 or 4 or 5 or 6 or 7 or 9

11. Dopamine Agonists/

12. dopamin* agonist*.ti.

13. cabergolin*.mp. or Cabergoline/

14. 11 or 12 or 13

15. 10 and 14

16. limit 15 to human

### DATABASE: WEB OF SCIENCE

# 8 #7 AND #6

Indexes=SCI-EXPANDED, SSCI, A&HCI, CPCI-S, CPCI-SSH, ESCI Timespan=All years

# 7 TOPIC: (cabergolin*)

Indexes=SCI-EXPANDED, SSCI, A&HCI, CPCI-S, CPCI-SSH, ESCI Timespan=All years

# 6 #5 OR #4 OR #3 OR #2 OR #1

Indexes=SCI-EXPANDED, SSCI, A&HCI, CPCI-S, CPCI-SSH, ESCI Timespan=All years

# 5 TOPIC: (Macro-prolactinoma*)

Indexes=SCI-EXPANDED, SSCI, A&HCI, CPCI-S, CPCI-SSH, ESCI Timespan=All years

# 4 TOPIC: (Macroprolactinoma*)

Indexes=SCI-EXPANDED, SSCI, A&HCI, CPCI-S, CPCI-SSH, ESCI Timespan=All years

# 3 TOPIC: (Micro-prolactinoma*)

Indexes=SCI-EXPANDED, SSCI, A&HCI, CPCI-S, CPCI-SSH, ESCI Timespan=All years

# 2 TOPIC: (Microprolactinoma*)

Indexes=SCI-EXPANDED, SSCI, A&HCI, CPCI-S, CPCI-SSH, ESCI Timespan=All years

# 1 TOPIC: (Prolactinoma*)

Indexes=SCI-EXPANDED, SSCI, A&HCI, CPCI-S, CPCI-SSH, ESCI Timespan=All years

### DATABASE: CINAHL EBSCO HOST

#### DATE OF SEARCH: 1974 TO JANUARY 7, 2021

S 1 (MH “Prolactinoma”) OR “Prolactinoma”

S 2 TI Prolactinoma* OR AB Prolactinoma* OR TX Micro-prolactinoma* OR TX Microprolactinoma* OR TX Macro-prolactinoma* OR TX Macroprolactinoma*

S 3 TX (prolactin* N3 (adenoma* OR carcinoma* OR tumor*))

S 4 TX PRL N5 (adenoma* OR carcinoma* OR tumor*))

S 5 S1 OR S2 OR S3 OR S4

S 6 (MH “Dopamine Agonists”)

S 7 TX Cabergolin*

S 8 TI dopamin* agonist*

S 9 S6 OR S7 OR S9

S 10 S5 AND S10

## Appendix 5

**Table:** EVALUATION OF METHODOLOGIC QUALITY OF INCLUDED STUDIES (CHECKLIST AMSTAR 2).

**Study**	**Item**																**Global evaluation**
	**1**	**2**	**3**	**4**	**5**	**6**	**7**	**8**	**9**	**10**	**11**	**12**	**13**	**14**	**15**	**16**	
Almalki, 2017	No	No	No	Prob.Yes	No	No	No	Yes	No	No	No MA	No MA	No	No	No	Yes	Critically low
Carijia, 2012	No	No	No	No	No	No	No	No	No	No	No MA	No MA	No	No	No	No	Critically low
D'Sylva, 2015	Yes	No	Yes	Yes	Yes	Yes	No	Yes	Prob.Yes	No	Yes	No	Yes	No	No	Yes	Critically low
dos Santos Nunes, 2011	Yes	No	Yes	Prob.Yes	Yes	Yes	No	Yes	No	No	Yes	No	No	No	No	Yes	Critically low
Huang, 2018	Yes	No	Yes	Yes	Yes	Yes	No	Yes	No	No	No MA	No MA	Yes	No	No	Yes	Critically low
Stiles, 2019	Yes	Prob.Yes	No	No	No	Yes	No	Prob.Yes	Prob.Yes	No	Yes	No	Yes	No	No	Yes	Critically low
Zamanipoor Najafabadi, 2020	No	No	No	Yes	Yes	Yes	No	Yes	No	No	Yes	Yes	Yes	No	No	Yes	Critically low

## AMSTAR CHECKLIST (73).

1. *Did the research questions and inclusion criteria for the review include the components of PICO?*

2. *Did the report of the review contain an explicit statement that the review methods were established prior to the conduct of the review and did the report justify any significant deviations from the protocol?*

3. *Did the review authors explain their selection of the study designs for inclusion in the review?*

4. *Did the review authors use a comprehensive literature search strategy?*

5. *Did the review authors perform study selection in duplicate?*

6. *Did the review authors perform data extraction in duplicate?*

7. *Did the review authors provide a list of excluded studies and justify the exclusions?*

8. *Did the review authors describe the included studies in adequate detail?*

9. *Did the review authors use a satisfactory technique for assessing the risk of bias in individual studies that were included in the review?*

10. *Did the review authors report on the sources of funding for the studies included in the review?*

11. *If meta-analysis (MA) was performed, did the review authors use appropriate methods for the statistical combination of results?*

12. *If meta-analysis was performed, did the review authors assess the potential impact of risk of bias in individual studies on the results of the meta-analysis or other evidence synthesis?*

13. *Did the review authors account for the risk of bias in primary studies when interpreting/discussing the results of the review?*

14. *Did the review authors provide a satisfactory explanation for, and discussion of, any heterogeneity observed in the results of the review?*

15. *If they performed quantitative synthesis, did the review authors carry out an adequate investigation of publication bias (small study bias) and discuss its likely impact on the results of the review?*

*Did the review authors report any potential sources of conflict of interest, including any funding they received for conducting the review?*
